# Supplementary material for: Towards unified AI-driven fracture mechanics: the extended deep energy method (XDEM)
Source: Nat Commun. 2026 Aug 15;17:8492. doi: 10.1038/s41467-026-76748-1 (PMC13484471; doi:10.1038/s41467-026-76748-1)
Supplement: Supplementary file 3 [file 41467_2026_76748_MOESM3_ESM.zip › pic/Appendix/Landscape/landscape_loads.pdf]

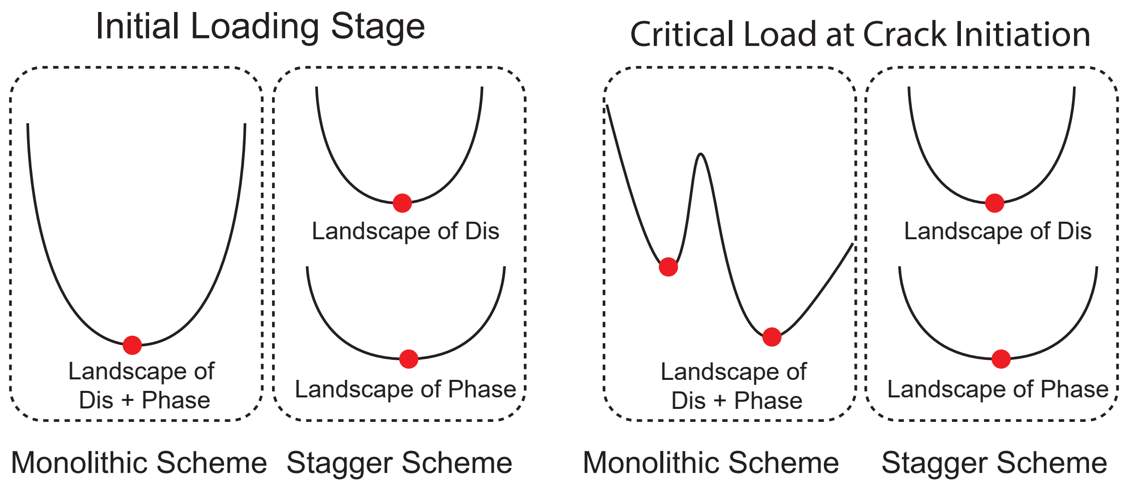
Initial Loading Stage
Critical Load at Crack Initiation
Landscape of Dis + Phase
Landscape of Dis
Landscape of Phase
Landscape of Dis + Phase
Landscape of Dis
Landscape of Phase
Monolithic Scheme
Stagger Scheme
Monolithic Scheme
Stagger Scheme
